# Public Heterogeneous Preferences for Low-Dose Computed Tomography Lung Cancer Screening Service Delivery in Western China: A Discrete Choice Experiment

**DOI:** 10.34172/ijhpm.8259

**Published:** 2024-07-10

**Authors:** Wenjuan Tao, Ting Bao, Tao Gu, Jay Pan, Weimin Li, Ruicen Li

**Affiliations:** ^1^Institute of Hospital Management, West China Hospital, Sichuan University, Chengdu, China; ^2^Health Management Center, General Practice Medical Center, West China Hospital, Sichuan University, Chengdu, China; ^3^School of Business Administration, Faculty of Business Administration, Southwestern University of Finance and Economics, Chengdu, China; ^4^HEOA Group, West China School of Public Health and West China Fourth Hospital, Sichuan University, Chengdu, China; ^5^School of Public Administration, Sichuan University, Chengdu, China; ^6^Department of Pulmonary and Critical Care Medicine, West China Hospital, Sichuan University, Chengdu, China; ^7^Institute of Respiratory Health, Frontiers Science Center for Disease-related Molecular Network, West China Hospital, Sichuan University, Chengdu, China; ^8^Precision Medicine Center, Precision Medicine Key Laboratory of Sichuan Province, West China Hospital, Sichuan University, Chengdu, China; ^9^State Key Laboratory of Respiratory Health and Multimorbidity, West China Hospital, Chengdu, China

**Keywords:** Discrete Choice Experiment, Heterogeneous Preferences, Lung Cancer Screening, Service Delivery

## Abstract

**Background:**

Lung cancer screening (LCS) with low-dose computed tomography (LDCT) is an efficient method that can reduce lung cancer mortality in high-risk individuals. However, few studies have attempted to measure the preferences for LDCT LCS service delivery. This study aimed to generate quantitative information on the Chinese population’s preferences for LDCT LCS service delivery.

**Methods:**

The general population aged 40 to 74 in the Sichuan province of China was invited to complete an online discrete choice experiment (DCE). The DCE required participants to answer 14 discrete choice questions comprising five attributes: facility levels, facility ownership, travel mode, travel time, and out-of-pocket cost. Choice data were analyzed using mixed logit and latent class logit (LCL) models.

**Results:**

The study included 2529 respondents, with 746 (29.5%) identified as being at risk for lung cancer. Mixed logit model (MLM) analysis revealed that all five attributes significantly influenced respondents’ choices. Facility levels had the highest relative importance (44.4%), followed by facility ownership (28.1%), while out-of-pocket cost had the lowest importance (6.4%). The at-risk group placed relatively more importance on price and facility ownership compared to the non-risk group. LCL model identified five distinct classes with varying preferences.

**Conclusion:**

This study revealed significant heterogeneity in preferences for LCS service attributes among the Chinese population, with facility level and facility ownership being the most important factors. The findings underscore the need for tailored strategies targeting different subgroup preferences to increase screening participation rates and improve early detection outcomes.

## Background

**Key Messages**

**Implications for policy makers**

- Expand access to high-quality lung cancer screening (LCS) services through tertiary hospital collaboration, such as establishing cross-level integrated healthcare networks or developing tele-health services with mobile screening vans.
- Implement targeted outreach and education campaigns tailored to the specific concerns and needs of different subgroups, such as addressing financial concerns and provider credibility for the at-risk group, and emphasizing service quality and convenience for the non-risk group.
- Adopt a segmented approach to service delivery, considering the distinct preferences and demographic characteristics of different classes identified in the study, such as addressing facility quality concerns for the opt-out classes (older and male individuals).
- Provide financial support options and incentives to address cost-related barriers, particularly for the at-risk and lower-income subgroups who placed greater emphasis on out-of-pocket costs.

**Implications for the public**

This research aimed to understand what factors matter most to you when it comes to lung cancer screening (LCS) services. We found that people have diverse preferences, but overall, they prioritize receiving high-quality services from reputable healthcare facilities. However, accessibility and affordability also play crucial roles in your willingness to participate in screening programs. By considering these preferences, policy-makers can tailor screening services to better meet your specific needs and concerns. For example, they could bring screening services closer to your communities, provide financial support options, and ensure convenient access. By addressing these factors, more people are likely to participate in LCS, leading to earlier detection and better health outcomes for all.

Lung cancer is the leading cause of cancer deaths worldwide.^1^ In China, approximately 0.73 million people died from lung cancer in 2022, accounting for 28.5% of all cancer deaths.^2^ About 67.4% of patients are diagnosed at a late stage,^3^ with only a 16.1% five-year survival rate in China.^4^ Early screening is essential for improving the prognosis of lung cancer.^5^ Low-dose computed tomography (LDCT) is an evidence-based, efficient screening method that can reduce lung cancer mortality in high-risk individuals and is widely recommended in guidelines.^6,7^ In 2012, the Chinese government launched the Cancer Screening Program in Urban China, which includes LDCT lung cancer screening (LCS) for high-risk individuals.^8^

 Although the Cancer Screening Program in Urban China had been incorporated into China’s National Major Medical Reform Special Project and the National Major Public Health Special Project, the uptake rate of LCS among high-risk individuals remains poor. The uptake rate ranged from only 31.92% in 2013 to 34.86% in 2018, lower than the rates for breast cancer and liver cancer.^9,10^ Improving cancer screening uptake depends on people’s willingness to participate. Therefore, it is important to understand population preferences regarding LDCT LCS. This understanding would help policy-makers implement effective screening programs.

 A commonly used method for exploring and quantifying preferences for health services is the discrete choice experiment (DCE). In a DCE, respondents choose among hypothetical scenarios (eg, screening options) described by a set of attributes, each with different levels combined to represent various choice alternatives. An increasing number of studies have examined preferences for cancer screening using DCEs. For instance, Norman et al^11^ and Zhao et al^12^ utilized DCEs in the context of LCS. They analyzed preferences of high-risk individual for LCS, focusing on attributes related to the screening test, such as screening tools, screening intervals, and radiation exposure.

 Access to screening (eg, travel distance, healthcare facilities availability) is one of the major barriers to LCS.^13,14^ Understanding the public’s preferences for service delivery attributes can help improve the delivery of preventive health services from a supply-side perspective. There is a need for additional studies on service delivery attributes and expanding the study participants to more public populations to improve cancer screening uptake.^15^ Furthermore, studies often assume respondents’ preferences are homogenous, although preferences may be heterogenous among individuals or groups within a given population.^16^ Ignoring preference heterogeneity may bias the utility estimates derived from the DCE study.^17,18^ It is important to consider preference heterogeneity in DCE data when interpreting evidence on service delivery preferences for LCS.

 This study aims to analyze the public heterogeneous preferences in China for LDCT LCS using a DCE, focusing on the features of service delivery attributes among both the at-risk and non-at-risk lung cancer populations. The findings could provide policy-makers with information to enhance LCS programs by improving service delivery, thereby increasing the screening uptake rate.

## Methods

 The reporting of this DCE referenced the checklist for conjoint analysis applications in health developed by the ISPOR Good Research Practices for Conjoint Analysis Task Force.^19^ This study was approved by the Ethics Committee of the authors’ institute.

###  Identification and Selection of Attributes and Levels

 The identification and selection of attributes were based on a step-wise approach. First, a systematic review of studies about population preferences for LCS and other cancer screenings was conducted to identify possible attributes. These attribute characteristics can be divided into Hall and colleagues’ framework (test-specific, outcomes, service delivery, and monetary)^20^ and Mandrik and colleagues’ framework (procedure-related, organization-related, and provider/population-related).^21^ Since our research aimed to understand residents’ preference for the service delivery of LCS, we chose a set of related attributes from the service delivery and organization-related dimensions. Second, in-depth qualitative interviews were conducted with people (n = 10) aged 40–74 at the Health Management Center of Leading/Teaching Hospital in Sichuan. Participants were asked to comment on the selected list of LCS attributes extracted from the literature and propose other possible attributes. Third, the multidisciplinary research team, including clinical experts, hospital managers, policy-makers, and economists, agreed on the final five attributes: facility levels, facility ownership, travel mode, travel time, and out-of-pocket cost. The final attributes mainly considered the facility, affordability, accessibility of the organization factors,^21^ as well as prior research.^11,12^

 The levels of each attribute were selected during the multidisciplinary research team consultation based on certain criteria. First, all levels should be plausible; second, they should fit the current Chinese context and practice; finally, they should be spread enough for respondents to trade-off between them, but not so spread that an option with the poorer level is never chosen.^11^ The facility levels and ownership were set according to the classification standards of the China Health Statistics Yearbook. When seeking care, travel mode often includes public transportation and private automobiles. Since preventive screening services are non-emergency medical services, and considering the way Chinese residents are accustomed to traveling, walking and bicycling were also included in this attribute. Travel time options ranging from <15 to >60 minutes were based on distances to the nearest LCS sites in the rural and urban geographic regions. It was considered that travel time could be shortest (less than 15 minutes) and longest (at least 60 minutes).^22^ The levels of the attribute “out-of-pocket cost” were set at 0, 150, 250, 350, and 450 CNY. Given that LDCT LCS is not covered by medical insurance, these levels were chosen to represent varying degrees of out-of-pocket expenses for participants. The final set of attributes and levels are reported in Table 1.

**Table 1:** Selected Attributes and Levels

**Attributes**	**Levels**	**Descriptions**
Facility levels	Primary healthcare institution First-level hospital Secondary hospital Tertiary hospital	Type of facility: where the screening was performed. Different levels of medical institutions have different service functions.*
Facility ownership	Public	Type of facility: where the screening was performed.
	Private	
Travel mode	Walking	Accessibility: the convenience of transportation. Public transportation includes bus and subway, etc.
	Bicycling	
	Public transportation	
	Private automobile	
Travel time (min)	<15	Accessibility: distance from home to the screening site.
	15-30	
	30-60	
	>60	
Out-of-pocket cost (CNY)	0	Affordability: screening costs. The cost of an ordinary CT examination in China is around 200 CNY.
	150	
	250	
	350	
	450	

*Note*. Exchange rate: 1 USD=6.98 CNY, December 7, 2022. “30 minutes” level refers to the 30-60 minutes time range. * Primary healthcare institutions include community health service centers and township hospitals. First-level hospitals are primary hospitals and health centers that directly provide preventive, medical, healthcare, and rehabilitation services to communities with a certain population, with the number of beds ≤100. Secondary hospitals are regional hospitals that provide medical and health services across several communities, with the number of beds ranging from 101 to 500. Tertiary hospitals are hospitals that provide medical and health services across regions, provinces, cities, and nationwide. They are medical technology centers with comprehensive medical, teaching, and scientific research capabilities, with the number of beds ≥501.

###  Choice Tasks and Experimental Design

 The experimental design, an orthogonal design, was generated using the dcreate command in the statistical software Stata 17.0. To minimize respondents’ cognitive burden and the risk of losing interest during the DCE task, consideration must be given to the number and complexity of choice tasks.^23^ According to the ISPOR Good Research Practices for Conjoint Analysis Task Force, the recommended number of choice sets is between 8 and 16.^19^ In our DCE design, the number of choice tasks per participant was set at 14. It was deemed a number low enough to avoid excessive cognitive load but high enough to establish sufficient statistical precision. Each choice set comprised two contrasted generic alternatives and a fixed “opt-out” alternative (“No screening”).

###  Questionnaire Design

 The questionnaire consisted of four parts (Supplementary file 1). First, related demographic and socioeconomic information was collected, such as sex, age, education level, and employment status. Age group and location were first asked to screen potential respondents aged 40-74 residing in Sichuan province. Previous experience with LCS was also asked about. Second, respondents were asked questions to identify their lung cancer risk factors according to the “China Guideline for the Screening and Early Detection of Lung Cancer” (hereinafter referred to as the “Guide”).^24^ Third, the DCE involved 14 choice tasks. Before the formal survey, we conducted a pre-survey to test how participants understand and perceive the questions, as well as attributes and levels descriptions. We modified some of the descriptive statements to ensure clarity.

###  Sampling and Data Collection

 Sample size calculations were based on the most cited rule of thumb and the equation N>500c/(t×a).^25^ The required sample size for the main effects depends on the number of choice tasks (t), the number of alternatives within each choice set (a), and the highest number of attribute levels across all attributes (c). Based on the final list of attributes and attribute levels, with t = 14, a = 3, and c = 5, the minimum required sample size was N = 60 respondents. To allow for subgroup analyses by lung cancer risk group and non-risk group (as about 21.34% of residents may be at high risk),^26^ the minimum sample size was increased to N = 282.

 The survey was conducted via a web-based platform (the questionnaires were completed using SurveyStar through WeChat) between August 27 and September 9, 2022, in Sichuan province, China. Sichuan is the largest economy in western China and can be divided into two distinctly different areas (eastern region and western region) similar to the whole of China, exhibiting a miniature of China.^27^ Survey participants were selected through stratified proportional sampling in combination with convenience sampling and snowball sampling. The sample distribution is shown in Table S1 of Supplementary file 2.

 Inclusion criteria include (1) residents who have lived in Sichuan province for at least one year; (2) asymptomatic individuals aged 40 to 74 with no history of lung cancer. Participation was voluntary based on informed consent, including the right to refuse or withdraw without any disadvantages. We provided a small financial incentive in the form of a WeChat red envelope (1-5 CNY) to respondents upon completion of the survey to encourage participation and attentiveness.

 After survey collection, we performed data quality checks for stated preference data. We examined the prevalence of always choosing the left-hand or right-hand alternative, always choosing the alternative with a better level of a single-ordered attribute (attribute dominance), rushing through the survey with short response times, and failing comprehension questions.^28^

###  Statistical Analysis

 Following the survey, the collected data were cleaned and converted to the dataset format required for statistical analyses. Descriptive statistics were conducted for the variables. Sociodemographic variables were presented as frequencies or percentages of categorical variables. The samples were classified into two groups (non-risk and risk groups) based on whether they had any of the lung cancer risk factors stated in the Guideline, which includes: (1) being an ever-smoker with more than 30 pack-years or quitting smoking within 15 years; (2) living with smokers for up to 20 years; (3) having a history of chronic obstructive pulmonary disease; (4) having experienced occupational exposure (eg, asbestos, radon, beryllium, uranium, chromium, etc) for at least 1 year; and (5) having a family history of lung cancer. Chi-square tests were used to assess the differences in characteristics between the non-risk and risk groups within the participating population.

 A mixed logit model (MLM) and a latent class logit (LCL) model were used to investigate preference heterogeneity among respondents. These two models are often used to complement each other in elucidating different aspects of preference heterogeneity and are applied in many preference analysis studies.^29,30^

 First, the MLM was used to observe preference and preference heterogeneity across individuals for LCS. The model provides mean coefficients as well as a measure of their distributions in the form of a standard deviation (SD). A statistically significant SD indicates that there is significant preference heterogeneity for that attribute.^31^ The subgroup analysis was conducted for both risk and non-risk groups by dividing the sample into two groups and estimating a MLM for each group. The assumption of a normal distribution is applied, and the number of draws used is 500.

 Further, the LCL was used to observe preference heterogeneity across groups (or classes). The optimal number of classes was determined by comparing the Bayesian information criterion (BIC) and consistent Akaike information criterion (CAIC) for LCL class solutions ranging from two to seven classes, with a lower value implying a better fit.^32^ Variables predicting the class membership include age, sex, education level, employment status, location, area, monthly income, lung cancer risk, and previous LCS experience.

 To overcome differences in scale for comparison, relative importance scores (RISs) of attributes were calculated based on model estimations to quantify the importance of each attribute relative to others. All attribute variables were coded as dummy variables, except for cost, which was modeled as a continuous variable. The value associated with the ‘opt-out’ choice is expressed as a constant, known as the alternative specific constant (ASC). All variables were dummy coded, except for age, income, area, and education level, which were treated as continuous variables. A two-sided *P* value <0.05 was considered to be statistically significant. All statistical analyses were conducted using Stata 17.0. (StataCorp, College Station, TX, USA).

## Results

###  Description of the Study Participants

 With invalid surveys excluded, a total of 2529 respondents were included in the final analysis. The geographical distribution of the respondents was representative of the Sichuan province (Supplementary file 2, Table S1). The average age of respondents was 49.57 ± 7.78 years, and more than half (57.49%) were female. The majority (79.04%) were urban residents, nearly half (47.93%) had a university education or above, and more than three-quarters (76.87%) were employed. Of the respondents, 32.08% had never been screened for lung cancer, 32.20% participated in LCS once a year, and 29.50% (n = 746) were at risk for lung cancer. There were significant differences in age, education, and employment characteristics between the lung cancer risk group and the non-risk group. The detailed self-reported characteristics of respondents are presented in Table 2.

**Table 2:** Self-reported Characteristics of Respondents (n = 2529)

**Characteristics**	**Total**	**Risk Group**	**Non-risk Group**	***P*** **Value**^b^
	**N = 2529 (%)**	**n = 746 (%)**	**n = 1783 (%)**	
Gender				.383
Female	1454 (57.49)	419 (56.17)	1035 (58.05)	
Male	1075 (42.51)	327 (43.83)	748 (41.95)	
Age (y)				.000
40~49	1328 (52.51)	300 (40.21)	1028 (57.66)	
50~59	924 (36.54)	337 (45.17)	587 (32.92)	
60~69	240 (9.49)	98 (13.14)	142 (7.96)	
70~74	37 (1.46)	11 (1.47)	26 (1.46)	
Location				.074
Urban	1999 (79.04)	573 (76.81)	1426 (79.98)	
Rural	530 (20.96)	173 (23.19)	357 (20.02)	
Areas^a^				
First class	689 (27.24)	202 (27.08)	487 (27.31)	.089
Second class	1663 (65.76)	479 (64.21)	1184 (66.40)	
Third class	177 (7.00)	65 (8.71)	112 (6.28)	
Education level				.000
Primary school or below	132 (5.22)	49 (6.57)	83 (4.66)	
Junior middle school	277 (10.95)	102 (13.67)	175 (9.81)	
High school	338 (13.36)	125 (16.76)	213 (11.95)	
Vocational diploma	570 (22.54)	177 (23.73)	393 (22.04)	
University or above	1212 (47.93)	293 (39.27)	919 (51.54)	
Employment				.000
Employed	1944 (76.87)	516 (69.17)	1428 (80.09)	
Not employed	163 (6.45)	56 (7.51)	107 (6.00)	
Retired	422 (16.69)	174 (23.32)	248 (13.91)	
Insurance				NA^c^
UEBMI	2098 (82.96)	609 (81.64)	1489 (83.51)	
URRMI	392 (15.50)	124 (16.62)	268 (15.03)	
CMI	260 (10.28)	70 (9.38)	190 (10.66)	
Uninsured	24 (0.95)	10 (1.34)	14 (0.79)	
Monthly income (CNY)				.712
≤3000	335 (13.25)	104 (13.94)	231 (12.96)	
3000-5000	490 (19.38)	140 (18.77)	350 (19.63)	
5000-8000	800 (31.63)	244 (32.71)	556 (31.18)	
≥8000	904 (35.75)	258 (34.58)	646 (36.23)	
Lung cancer screening status				
Never screened	819 (32.08)	239 (32.04)	573 (32.14)	.065
Ever screened, not up to date	748 (29.30)	194 (26.01)	547 (30.68)	
Once a year	822 (32.20)	259 (34.72)	556 (31.18)	
I don't know	164 (6.42)	54 (7.24)	107 (6.00)	
Lung cancer risk factors				
Smoking	111 (4.39)	111 (14.88)		
Second-hand smoking	333 (13.17)	333 (44.66)		
With chronic obstructive pulmonary disease	34 (1.34)	34 (4.56)		
Occupational exposure to carcinogens	119 (4.71)	119 (15.95)		
Family history	312 (12.34)	312 (41.82)		

Abbreviations: UEBMI, Urban Employee Basic Medical Insurance; URRMI, Urban and Rural Resident Medical Insurance; CMI, Commercial Medical Insurance. ^a^ According to the “Sichuan Health Statistical Yearbook,” there are three types of regions categorized according to resource allocation: the first to third class represents the degree of healthcare resources from high to low. ^b^ Chi-square test with a significance level of *P *≤.05. ^c^ The chi-square value cannot be calculated because of multiple choices. Residents may have both basic medical insurance (UEBMI or URRMI) and supplementary medical insurance (CMI).

###  Mixed Logit Model

 The MLM was adopted to analyze the preferences for LCS service delivery with LDCT and heterogeneity differences in individual preferences. All five attributes included in the analysis significantly influenced the respondents’ choices of alternatives (*P*< .05). The analysis of the full sample showed that the overall population did not have a significant preference for or against engaging in the screening behavior (the ASC coefficient was insignificant). The preference of the no-risk group was consistent with that of the overall population, while the at-risk group exhibited a higher preference for the screening behavior. The two groups’ preferences for the individual attributes in terms of direction and magnitude were generally similar to those of the overall population, preferring services that were located at tertiary public hospitals, reachable by walking or cycling, with a duration of 15–30 minutes, and at a lower cost. Almost all SDs of the attribute levels were statistically significant, indicating preference heterogeneity across respondents. The results of the MLM model are reported in Table 3.

**Table 3:** Results of Mixed Logit Analysis

**Attributes and Levels**	**Risk Group**		**Non-risk Group**		**All**	
	**Coefficient (SE)**	**SD (SE)**	**Coefficient (SE)**	**SD (SE)**	**Coefficient (SE)**	**SD (SE)**
ASC	1.353** (0.229)	4.819** (0.214)	0.053 (0.143)	4.849** (0.110)	0.034 (0.129)	4.849** (0.110)
Facility levels						
PHCI (base)						
First-level hospital	0.426** (0.063)	0.069 (0.140)	0.458** (0.042)	0.236** (0.063)	0.457** (0.036)	0.236** (0.063)
Secondary hospital	0.703** (0.074)	0.513** (0.120)	0.736** (0.051)	0.581** (0.064)	0.737** (0.042)	0.581** (0.064)
Tertiary hospital	3.112** (0.118)	2.033** (0.095)	3.304** (0.078)	2.294** (0.056)	3.252** (0.064)	2.294** (0.056)
Facility ownership						
Private (base)						
Public	2.098** (0.085)	1.540** (0.075)	2.027** (0.054)	1.656** (0.044)	2.054** (0.045)	1.656** (0.044)
Travel mode						
Walking (base)						
Bicycling	0.061 (0.067)	0.396** (0.129)	0.106* (0.045)	0.584** (0.051)	0.081* (0.038)	0.584** (0.051)
Public transportation	-0.503** (0.072)	-0.763** (0.098)	-0.541** (0.048)	0.861** (0.053)	-0.535** (0.041)	0.861** (0.053)
Private automobile	-0.350** (0.066)	-0.166 (0.143)	-0.236** (0.042)	0.187* (0.078)	-0.276** (0.036)	0.187* (0.078)
Travel time (min)						
<15 (base)						
15-30	0.516** (0.073)	0.045 (0.102)	0.518** (0.047)	0.013 (0.059)	0.515** (0.040)	0.013 (0.059)
30-60	-0.031 (0.064)	0.014 (0.095)	-0.067 (0.042)	0.004 (0.059)	-0.056 (0.035)	0.004 (0.059)
>60	-0.328** (0.078)	0.661** (0.098)	-0.452** (0.053)	0.783** (0.053)	-0.410** (0.044)	0.783** (0.053)
Out-of-pocket cost	-0.001** (0.000)	0.004** (0.000)	-0.001** (0.000)	0.003 (0.000)	-0.001** (0.000)	0.003** (0.000)
Log likehood	-6502.929		-15528.25		-22 018.232	
AIC	130 53.86		31 104.50		44 084.46	
BIC	132 54.31		31 325.87		44 314.22	
Number of respondents	746		1783		2529	
Number of observations	31 332		74 886		106 218	

Abbreviations: PHCI, primary healthcare institution; SE, standard error; SD, standard deviation; AIC, Akaike information criterion; BIC, Bayesian information criterion; ASC, alternative specific constant. *Note*. Significance levels ** *P *< .01; * *P *< .05.

 According to the overall RIS, the attribute “facility levels” had the highest RIS of 44.4%, followed by “facility ownership” at 28.1%, while “out-of-pocket cost” had the lowest score of 6.4%. The results of the RIS for the subgroups showed that the order of importance did not differ much between the two groups, but the magnitudes did vary. The at-risk group placed relatively more importance on price and facility ownership compared to the no-risk group, whereas the no-risk group placed relatively more importance on facility levels, mode of access, and duration compared to the at-risk group. The results of RIS are shown in Figure.

**Figure:**
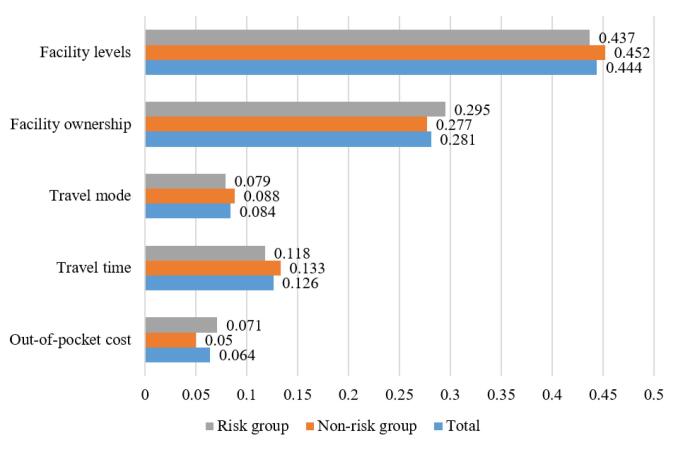
Relative Importance Score of Mixed Logit Analysis.

###  Latent Class Logit Model

 We used LCL to understand heterogeneity and identified five classes with different preferences based on BIC and CAIC (See Supplementary file 2, Table S3 and Figure S1). Based on the results of the latent class model (Table 4), there were essentially five groups (classes) of participants. Class 1 prefers to opt out, comprising 29.3% of the total population, and this class is insensitive to the price attribute. Class 2 prefers to choose the provided screening program, and this class finds higher-priced programs more attractive, accounting for 22.9% of the total population. Class 3 also tends to opt out and prefers to access the healthcare facility by private car, comprising 16.1% of the total population. Class 4 prefers to choose the provided screening program and tends to prefer accessing the facility by walking. This class is also insensitive to the service price, accounting for 19.8% of the total population. Class 5 demands shorter durations and is most sensitive to price, comprising 12.0% of the total population.

**Table 4:** Results of Latent Class Logit Analysis

**Attributes and Levels**	**Class 1**		**Class 2**		**Class 3**		**Class 4**		**Class 5**	
	**Coefficient **	**SE**	**Coefficient **	**SE**	**Coefficient **	**SE**	**Coefficient **	**SE**	**Coefficient**	**SE**
ASC	-5.928**	0.261	2.511**	0.257	-1.674**	0.130	1.682**	0.272	3.124**	0.159
Facility levels										
PHCI (base)										
First-level hospital	1.043**	0.184	0.764**	0.071	0.375**	0.083	-0.119	0.145	0.239**	0.065
Secondary hospital	0.724**	0.199	1.795**	0.149	1.068**	0.082	0.705**	0.198	0.179**	0.066
Tertiary hospital	5.096**	0.178	3.104**	0.141	2.168**	0.092	1.093**	0.161	0.135	0.077
Facility ownership										
Private (base)										
Public	1.872**	0.099	1.214**	0.066	1.159**	0.053	3.463**	0.108	0.244**	0.040
Travel mode										
Walking (base)										
Bicycling	0.543**	0.114	0.425**	0.071	0.136	0.074	-0.374**	0.128	-0.131*	0.061
Public transportation	0.130	0.123	-1.451**	0.087	0.094	0.075	-0.360*	0.144	0.253**	0.069
Private automobile	0.658**	0.102	-0.615**	0.067	0.281**	0.069	-0.270*	0.137	-0.112	0.063
Travel time (min)										
<15 (base)										
15-30	0.560**	0.128	0.460**	0.076	0.178*	0.079	-0.080	0.131	-0.186**	0.067
30-60	-0.458**	0.103	0.218**	0.073	-0.030	0.070	0.255	0.135	-0.274**	0.064
>60	-0.296**	0.109	-1.418**	0.114	-0.024	0.082	-1.008**	0.165	-0.267**	0.071
Out-of-pocket cost	-0.000	0.000	0.002**	0.000	-0.001**	0.000	-0.001	0.000	-0.002**	0.000
Average class shares	0.293		0.229		0.161		0.198		0.120	
Class membership model parameters: Class 5 = Reference class										
Age	0.048**	0.013	0.013	0.014	0.032*	0.015	0.013	0.014		
Sex (base: female)	0.310*	0.157	-0.040	0.164	0.497**	0.179	-0.005	0.163		
Education										
Primary school or below (base)										
Junior middle school	0.167	0.462	-0.443	0.389	-0.757	0.404	-0.142	0.386		
High school	0.524	0.460	-0.438	0.397	-0.952*	0.417	-0.144	0.393		
Vocational diploma	0.817	0.458	-0.184	0.393	-0.776	0.411	-0.032	0.395		
University or above	1.409**	0.461	-0.019	0.400	-0.383	0.415	-0.315	0.404		
Employment										
Employed (base)										
Not employed	0.150	0.315	-0.267	0.309	-0.862*	0.369	-0.206	0.301		
Retired	0.401	0.302	0.703*	0.312	0.404	0.331	0.488	0.308		
Location (base: rural)	0.363	0.191	0.037	0.190	-0.162	0.200	0.220	0.191		
Areas										
First class (base)										
Second class	-0.349	0.188	-0.044	0.205	-0.123	0.215	-0.051	0.200		
Third class	-0.760*	0.340	-0.290	0.350	-0.192	0.369	0.044	0.328		
Income	0.285**	0.085	0.062	0.087	0.097	0.093	0.096	0.086		
Lung cancer risk	-0.375*	0.166	-0.373*	0.174	-0.370*	0.186	-0.127	0.170		
Lung cancer screening										
Never screened (base)										
Ever screened, not up to date	0.068	0.187	-0.129	0.193	-0.386	0.208	-0.120	0.189		
Once a year	0.732**	0.215	0.465*	0.227	0.203	0.240	0.275	0.227		
I don't know	0.225	0.362	0.397	0.355	0.419	0.370	0.371	0.351		
_cons	-4.369**	0.941	0.020	0.916	-1.185	0.975	-0.619	0.911		

Abbreviations: PHCI, primary healthcare institution; SE, standard error; ASC, alternative specific constant. *Note*. Significance levels ** *P *< .01; * *P *< .05.

 According to the analysis of RIS from the latent class model (Table 5), each class attaches different levels of importance to the various attributes. Class 1 places the highest importance on facility levels, followed by facility ownership, and the least importance on out-of-pocket cost. Class 2 places the highest importance on facility levels, followed by travel time, and the least importance on out-of-pocket cost. Class 3 places the highest importance on facility levels, followed by facility ownership, and the least importance on travel time. Class 4 places the highest importance on facility ownership, followed by travel time, and the least importance on out-of-pocket cost. Class 5 places the highest importance on out-of-pocket cost, followed by travel mode, and the least importance on facility levels.

**Table 5:** Relative Importance Score of Latent Class Logit Analysis

**Attributes**	**Class 1**		**Class 2**		**Class 3**		**Class 4**		**Class 5**	
	**RIS**	**Rank**	**RIS**	**Rank**	**RIS**	**Rank**	**RIS**	**Rank**	**RIS**	**Rank**
Facility levels	0.589	1	0.351	1	0.494	1	0.185	3	0.118	5
Facility ownership	0.216	2	0.137	4	0.264	2	0.529	1	0.120	4
Travel mode	0.076	4	0.212	3	0.064	4	0.057	4	0.189	2
Travel time	0.118	3	0.212	2	0.047	5	0.193	2	0.135	3
Out-of-pocket cost	0.001	5	0.088	5	0.130	3	0.035	5	0.438	1

Abbreviation: RIS, relative importance score.

 Relative to Class 5, Class 1 members tended to be older, male, with higher education at the university level or above, residing in areas with higher healthcare resources, having non-risk lung cancer status, and undergoing screening once a year. Class 2 members tended to be retired, have non-risk lung cancer status, and undergo screening once a year. Class 3 members who preferred to choose the screening were more likely to be older and male.

## Discussion

###  Principle Findings

 In this study, we conducted a DCE to investigate the preferences and heterogeneity in preferences among the Chinese population for LDCT LCS service delivery attributes. The results revealed significant heterogeneity in preferences across respondents, with distinct subgroups exhibiting varying levels of importance for different attributes. This study yielded the following major findings:

 First, the MLM analysis revealed that all five attributes significantly influenced the respondents’ choices. The attribute “facility levels” was the most important factor compared to other factors, followed by “facility ownership,” while “out-of-pocket cost” had the lowest relative importance. This finding differs from an Australian study where the type of facility for LCS did not appear to matter once distance was controlled.^11^ Prior evidence suggested that the accessibility of preventive healthcare facilities plays a key role in the decision to undergo cancer screening.^33^ Our study finding indicated that Chinese residents are willing to sacrifice part of the accessibility and choose high-level public hospitals for LCS. Possible explanations for this finding may include: First, it can be interpreted as reflecting respondents’ care-seeking behavior or habit with the healthcare system in China. Due to long-term unbalanced resource allocation and differences in healthcare service capabilities, Chinese patients are more likely to trust high-level hospitals and crowd into tertiary hospitals.^34,35^ Second, the Chinese healthcare delivery system is “mixed” with a dominant role for public healthcare institutions. Chinese people showed a high preference for obtaining healthcare from public providers.^36^

 Second, the results of the subgroup analysis showed that: (1) The at-risk group showed a higher preference for the screening program compared to the no-risk group. This difference in preferences may be attributed to the heightened awareness and perceived need for screening among individuals with risk factors for lung cancer. (2) Both groups generally preferred services located at tertiary public hospitals, reachable by walking or cycling, with a duration of 15–30 minutes, and at a lower cost. It suggests a high level of trust in the quality and capabilities of these facilities and the importance of convenient access in promoting participation. (3) The at-risk and no-risk groups diverged in their relative importance placed on certain attributes. The at-risk group placed more emphasis on price and facility ownership, potentially reflecting financial concerns and a desire for reputable healthcare providers given their higher perceived risk. In contrast, the no-risk group prioritized facility levels, travel mode and travel time, suggesting a greater focus on service quality and convenience when perceived risk is lower.

 Third, the LCL analysis identified five distinct classes of respondents with varying preferences and demographic characteristics for LCS service attributes. It highlights the significant heterogeneity that exists within the population regarding their priorities and decision-making factors related to screening services. Notably, Classes 1 and 3 comprising 45.4% of the respondents, exhibited a preference for opting out of the LCS program altogether. Interestingly, these two classes placed the highest importance on the facility levels attribute, suggesting that they prioritized receiving services from high-quality healthcare facilities, potentially due to concerns about the reliability and expertise associated with different facility types. In terms of the demographic characteristics, both Classes 1 and 3 tended to comprise older and male individuals. This finding aligns with previous research indicating that older adults and men may be less likely to participate in preventive health services, potentially due to factors such as perceived risk, health beliefs, or access barriers.^37,38^

###  Strengths and Limitations

 A key strength of this study is the use of DCE and advanced statistical models (MLM and LCL) to quantify preferences and heterogeneity. The large sample size and representative geographical distribution of respondents from Sichuan province enhance the generalizability of the findings. Additionally, the inclusion of both at-risk and non-risk groups for lung cancer allowed for subgroup comparisons.

 However, the study has some limitations. First, the participants were mainly urban residents with higher education levels, as comprehending the DCE tasks online required a certain level of education. To reduce potential selection bias, we have taken measures such as broadening the sample range and employing stratified sampling techniques. Second, the study focused on a specific region (Sichuan), and preferences may vary across different cultural contexts. Nevertheless, the general pattern of findings could be useful guidance for other similar conditions. Third, travel mode and travel time may have some correlation. However, in reality, these two factors are closely related and important in influencing individuals’ screening choices. Treating them as completely independent attributes in the study may reduce the external validity of the results. Future research could further explore estimating the interaction between travel mode and travel time.

## Implications for Practice

 Based on the major findings of this study, here are the key implications for practice:

 First, to meet the public’s preference for high-quality services from tertiary hospitals while improving accessibility, it would be to expand access to high-quality LCS services through tertiary hospital collaboration. Potential strategies could be involved: (1) Establishing cross-level integrated healthcare networks, tertiary hospitals can provide training, quality control, and technical support to enable primary care facilities to offer reliable LCS services under their guidance. (2) Developing tele-health services, tertiary hospitals deploy mobile screening vans equipped with LDCT scanners and staffed by specialized personnel to periodically conduct screenings in communities.^39^

 Second, implementing targeted outreach and education campaigns is recommended. The subgroup analysis revealed differences in preferences between the at-risk and non-risk groups, with the former placing greater emphasis on cost and facility ownership. Tailored outreach and education campaigns should be developed to address the specific concerns and needs of these groups. For the at-risk group, campaigns could focus on available financial support options and the credibility of public healthcare providers. For the non-risk group, emphasis could be placed on promoting the quality of services and convenience factors.

 Third, the identification of five distinct classes with varying preferences and demographic characteristics highlights the need for a segmented approach to service delivery. Healthcare providers and policy-makers should consider tailoring their strategies to cater to the specific priorities and needs of each segment. For instance, for the opt-out classes (older and male individuals), efforts could be directed toward addressing their concerns regarding facility quality and expertise, potentially through targeted awareness campaigns or incentives. Additionally, removing access barriers and providing convenient service options may be crucial for promoting participation among these segments.

## Conclusion

 This study employed a DCE to investigate preferences for LCS service delivery attributes using LDCT among the Chinese population. The findings revealed significant heterogeneity in preferences across respondents, with distinct subgroups exhibiting varying levels of importance for different attributes. Overall, facility level was the most important attribute, followed by facility ownership, while out-of-pocket cost had the lowest relative importance. The study underscores the need to tailor service delivery strategies to address the diverse preferences and characteristics of different subgroups to increase screening participation rates, and ultimately improve population-level lung cancer prevention and early detection outcomes.

## Acknowledgements

 We thank the members of the HEOA Group who provided comments and suggestions on the questionnaire revision, and the health care professionals who participated in the DCE study. We are grateful to all the respondents who participated in the interviews and survey, as well as the ones who assisted with participant recruitment in the web.

## Ethical issues

 This study was approved by the Ethics Committee of West China Hospital in Sichuan University.

## Competing interests

 Authors declare that they have no competing interests.

## Supplementary files


Supplementary file 1. DCE Survey Instrument.

Supplementary file 2. contains Tables S1-S3 and Figure S1.
