# Supplementary material for: Public Heterogeneous Preferences for Low-Dose Computed Tomography Lung Cancer Screening Service Delivery in Western China: A Discrete Choice Experiment
Source: Int J Health Policy Manag. 2024 Jul 10;13:8259. doi: 10.34172/ijhpm.8259 (PMC11369360; doi:10.34172/ijhpm.8259)
Supplement: Supplementary file 1 — DCE Survey Instrument. [file ijhpm-13-8259-s001.pdf]

Article title: Public Heterogeneous Preferences for Low-Dose Computed Tomography Lung Cancer Screening Service Delivery in Western China: A Discrete Choice Experime

Journal name: International Journal of Health Policy and Management (IJHPM)

Authors' information: Wenjuan Tao[1], Ting Bao[2], Tao Gu[1,3], Jay Pan[4,5], Weimin Li[6,7,8,9], Ruicen Li[2]*

[1]Institute of Hospital Management, West China Hospital, Sichuan University, Chengdu, China.

[2]Health Management Center, General Practice Medical Center, West China Hospital, Sichuan University, Chengdu, China.

[3]School of Business Administration, Faculty of Business Administration, Southwestern University of Finance and Economics, Chengdu, China.

[4]HEOA Group, West China School of Public Health and West China Fourth Hospital, Sichuan University, Chengdu, China.

[5]School of Public Administration, Sichuan University, Chengdu, China.

[6]Department of Pulmonary and Critical Care Medicine, West China Hospital, Sichuan University, Chengdu, China.

[7]Institute of Respiratory Health, Frontiers Science Center for Disease-related Molecular Network, West China Hospital, Sichuan University, Chengdu, China.

[8]Precision Medicine Center, Precision Medicine Key Laboratory of Sichuan Province, West China Hospital, Sichuan University, Chengdu, China.

[9]State Key Laboratory of Respiratory Health and Multimorbidity, West China Hospital, Chengdu, China.

***Correspondence to:** Ruicen Li; Email: 991701168@qq.com

Citation: Tao W, Bao T, Gu T, Pan J, Li W, Li R. Public heterogeneous preferences for low-dose computed tomography lung cancer screening service delivery in western China: a discrete choice experiment. Int J Health Policy Manag. 2024;13:8259. doi:10.34172/ijhpm.8259

Supplementary file 1. DCE Survey Instrument

Part A: Participant characteristics

1. Are you:

   □ Male □ Female

2. What is your age: __________ years
3. Your current address:

   ________City _______District (County) ______Street (Town)

4. What is the highest level of education you have completed?

   □ Primary school or below

   □ Junior middle school

   □ High school

   □ Vocational diploma

   □ University or above

5. Which best describes your employment status?

   □ Employed

   □ Not employed

   □ Retired

6. What's your health insurance?

   □ Urban Employee Basic Medical Insurance

   □ Urban and Rural Resident Medical Insurance

   □ Commercial Medical Insurance

   □ Uninsured

7. What is your household monthly income in the last year?

   □ ≤3000 yuan

   □ 3000-5000 yuan

   □ 5000-8000 yuan

   □ ≥8000 yuan

8. Have you ever had a CT screening for lung cancer?

□ Never screened

□ Ever screened, not up to date

□ Once a year

□ I don't know

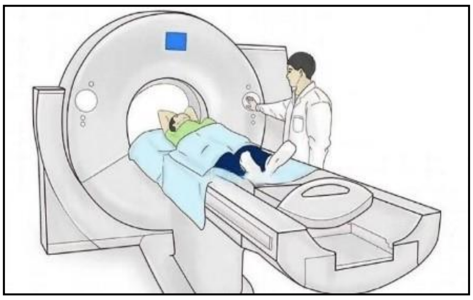

Part B: Lung cancer risk factors

1. Do you smoke:

   □ No (skip to question B7)

   □ Yes ("Those who have smoked for 6 months or more continuously or accumulatively in their lifetime" are defined as smokers)

2. Cumulative smoking time: ________ years (if there is a history of quitting smoking halfway, but relapses, the time limit for smoking cessation should be excluded)
3. Daily smoking amount: average _______ sticks/day
4. Are you currently quitting smoking?

   □ Yes (referring to those who have not smoked for more than 1 year, if they relapse, they have not quit smoking)

   □ No (please skip to question B7)

5. Years of quitting smoking: _______years
6. Daily smoking volume before quitting: average_______ sticks/day
7. Do you have long-term passive smoking? (Secondhand smoke is present for more than 15 minutes when you are not smoking)

   □ No

   □ Yes, have you lived or worked with a smoker for _____ years?

8. Have you been diagnosed with a chronic respiratory disease?

   □ No (skip to question B10)

   □ Yes

9. Which of the following chronic respiratory conditions have you been diagnosed with? [multiple choice]

□ Chronic Obstructive Pulmonary Disease

□ Bronchial Asthma

□ Pulmonary Fibrosis

□ Other ____________

10. Have you been exposed to harmful substances such as dust, soot, and chemical raw materials for a long time?

    □ No

    □ Yes, have you been exposed to dust, soot, chemical raw materials and other harmful substances for_____ years?

11. Have you ever had a first-degree relative (parent, child, half-sibling) diagnosed with lung cancer?

    □ Yes

    □ No

12. Have you ever had a first-degree relative (parent, child, sibling) diagnosed with lung cancer?

    □ Yes

    □ No

13. Have any other relatives in your family been diagnosed with malignant tumors:

    □ Yes, diagnosed as _________

    □ No

Part C: DCE Task

Imagine yourself in the situation described at the start.

Your doctor suggests that you may need a low-dose CT to check for lung cancer, so you decide to go to a hospital for lung cancer screening. You are given two different options for screening, or choosing not to screen.

Which of these services would you choose to take a lung cancer screening? (Option A, Option B or No screening)

Note:

Facility levels: Primary health care institutions: community health service centers and township hospitals; First-level hospitals: primary hospitals and health centers that directly provide preventive, medical, health care, and rehabilitation services to communities with a certain population, and the number of beds is ≤ 100; Secondary hospitals: regional hospitals that provide medical and health services across several communities, with beds ranging from 101 to 500; Tertiary hospitals: hospitals that provide medical and health services across regions, provinces, cities, and nationwide. They are medical technology centers with comprehensive medical, teaching, and scientific research capabilities, and the number of beds is ≥ 501.

Travel mode: Bicycling includes bicycle, electric bicycle and motorcycle; Public transportation includes bus and subway, etc. Private automobile includes self-driving car, taxi, online car-hailing.

Travel time: distance from home to the screening site.

(Please tick you preferred option)

Scenario 1

	**Option A□**	**Option B□**	**No screening□**
Facility levels	Tertiary hospital	Secondary hospital	
Facility ownership	Public	Private	
Travel mode	Public transportation	Private automobile	
Travel time	15-30 mins	<15 mins	
Out-of-pocket cost	150 yuan	0 yuan	

Scenario 2

	Option A□	Option B□	No screening□
Facility levels	Tertiary hospital	First-level hospital	
Facility ownership	Private	Public	
Travel mode	Walking	Bicycling	
Travel time	15-30 mins	>60 mins	
Out-of-pocket cost	0 yuan	450 yuan	

Scenario 3

	Option A□	Option B□	No screening□
Facility levels	First-level hospital	Primary health care institution	
Facility ownership	Public	Private	
Travel mode	Private automobile	Bicycling	
Travel time	30-60 mins	>60 mins	
Out-of-pocket cost	450 yuan	350 yuan	

Scenario 4

	Option A□	Option B□	No screening□
Facility levels	Tertiary hospital	First-level hospital	
Facility ownership	Public	Private	
Travel mode	Walking	Private automobile	
Travel time	>60 mins	15-30 mins	
Out-of-pocket cost	450 yuan	150 yuan	

Scenario 5

	**Option A□**	**Option B□**	**No screening□**
Facility levels	Primary health care institution	Secondary hospital	
Facility ownership	Public	Private	
Travel mode	Bicycling	Walking	
Travel time	<15mins	>60 mins	
Out-of-pocket cost	0 yuan	250 yuan	

Scenario 6

	**Option A□**	**Option B□**	**No screening□**
Facility levels	First-level hospital	Secondary hospital	
Facility ownership	Private	Public	
Travel mode	Bicycling	Private automobile	
Travel time	30-60 mins	>60 mins	
Out-of-pocket cost	250 yuan	150 yuan	

Scenario 7

	**Option A□**	**Option B□**	**No screening□**
Facility levels	Tertiary hospital	Primary health care institution	
Facility ownership	Public	Private	
Travel mode	Bicycling	Walking	
Travel time	>60 mins	<15 mins	
Out-of-pocket cost	0 yuan	450 yuan	

Scenario 8

	Option A□	Option B□	No screening□
Facility levels	First-level hospital	Secondary hospital	
Facility ownership	Public	Private	
Travel mode	Walking	Public transportation	
Travel time	30-60 mins	15-30 mins	
Out-of-pocket cost	150 yuan	450 yuan	

Scenario 9

	Option A□	Option B□	No screening□
Facility levels	Primary health care institution	First-level hospital	
Facility ownership	Private	Public	
Travel mode	Walking	Public transportation	
Travel time	30-60 mins	<15 mins	
Out-of-pocket cost	450 yuan	350 yuan	

Scenario 10

	Option A□	Option B□	No screening□
Facility levels	Tertiary hospital	Primary health care institution	
Facility ownership	Private	Public	
Travel mode	Bicycling	Private automobile	
Travel time	<15 mins	15-30 mins	
Out-of-pocket cost	150 yuan	350 yuan	

Scenario 11

	Option A□	Option B□	No screening□
Facility levels	Secondary hospital	Tertiary hospital	
Facility ownership	Public	Private	
Travel mode	Walking	Private automobile	
Travel time	15-30 mins	30-60 mins	
Out-of-pocket cost	250 yuan	350 yuan	

Scenario 12

	Option A□	Option B□	No screening□
Facility levels	First-level hospital	Primary health care institution	
Facility ownership	Private	Public	
Travel mode	Walking	Public transportation	
Travel time	15-30 mins	30-60 mins	
Out-of-pocket cost	450 yuan	250 yuan	

Scenario 13

	Option A□	Option B□	No screening□
Facility levels	Secondary hospital	Primary health care institution	
Facility ownership	Public	Private	
Travel mode	Bicycling	Public transportation	
Travel time	30-60 mins	>60 mins	
Out-of-pocket cost	350 yuan	0 yuan	

Scenario 14

	**Option A□**	**Option B□**	**No screening□**
Facility levels	Tertiary hospital	Secondary hospital	
Facility ownership	Public	Private	
Travel mode	Private automobile	Public transportation	
Travel time	<15 mins	30-60 mins	
Out-of-pocket cost	250 yuan	0 yuan	
