# Supplementary material for: Public Heterogeneous Preferences for Low-Dose Computed Tomography Lung Cancer Screening Service Delivery in Western China: A Discrete Choice Experiment
Source: Int J Health Policy Manag. 2024 Jul 10;13:8259. doi: 10.34172/ijhpm.8259 (PMC11369360; doi:10.34172/ijhpm.8259)
Supplement: Supplementary file 2 — contains Tables S1-S3 and Figure S1. [file ijhpm-13-8259-s002.pdf]

Article title: Public Heterogeneous Preferences for Low-Dose Computed Tomography Lung Cancer Screening Service Delivery in Western China: A Discrete Choice Experime

Journal name: International Journal of Health Policy and Management (IJHPM)

Authors' information: Wenjuan Tao[1], Ting Bao[2], Tao Gu[1,3], Jay Pan[4,5], Weimin Li[6,7,8,9], Ruicen Li[2]*

[1]Institute of Hospital Management, West China Hospital, Sichuan University, Chengdu, China.

[2]Health Management Center, General Practice Medical Center, West China Hospital, Sichuan University, Chengdu, China.

[3]School of Business Administration, Faculty of Business Administration, Southwestern University of Finance and Economics, Chengdu, China.

[4]HEOA Group, West China School of Public Health and West China Fourth Hospital, Sichuan University, Chengdu, China.

[5]School of Public Administration, Sichuan University, Chengdu, China.

[6]Department of Pulmonary and Critical Care Medicine, West China Hospital, Sichuan University, Chengdu, China.

[7]Institute of Respiratory Health, Frontiers Science Center for Disease-related Molecular Network, West China Hospital, Sichuan University, Chengdu, China.

[8]Precision Medicine Center, Precision Medicine Key Laboratory of Sichuan Province, West China Hospital, Sichuan University, Chengdu, China.

[9]State Key Laboratory of Respiratory Health and Multimorbidity, West China Hospital, Chengdu, China.

***Correspondence to:** Ruicen Li; Email: 991701168@qq.com

Citation: Tao W, Bao T, Gu T, Pan J, Li W, Li R. Public heterogeneous preferences for low-dose computed tomography lung cancer screening service delivery in western China: a discrete choice experiment. Int J Health Policy Manag. 2024;13:8259. doi:10.34172/ijhpm.8259

Supplementary file 2

Table S1 Distribution of the number of respondents in each city of Sichuan Province

City	Population (million)	Population proportion	Minimum sample size	Number of questionnaires
Chengdu	20.938	0.2502	71	664
Zigong	2.489	0.0297	8	51
Panzhihua	1.212	0.0145	4	25
Luzhou	4.254	0.0508	14	101
Deyang	3.456	0.0413	12	83
Mianyang	4.868	0.0582	16	125
Guangyuan	2.306	0.0276	8	62
Suining	2.814	0.0336	9	77
Neijiang	3.141	0.0375	11	71
Leshan	3.16	0.0378	11	175
Nanchong	5.608	0.0670	19	141
Meishan	2.955	0.0353	10	75

Yibin	4.589	0.0548	15	270
Guang'an	3.255	0.0389	11	109
Dazhou	5.385	0.0644	18	141
Ya’an	1.435	0.0171	5	66
Bazhong	2.713	0.0324	9	71
Ziyang	2.309	0.0276	8	45
A’ba	0.823	0.0098	3	19
Ganzi	1.107	0.0132	4	30
Liangshan	4.858	0.0581	16	128
Total	83.675	1.0000	282	2529

Note. Data source of “Population” is from Sichuan Provincial Health Statistical Yearbook (2020). Stratified proportional sampling is used to calculate the minimum sample size of each city in Sichuan Province.

Table S2. DCE design

Choice task	Alternative	Constant	Facility levels			Facility ownership	Travel mode			Travel time			Out-of-pocket cost
		A	B1	B2	B3	C1	D1	D2	D3	E1	E2	E3	F
1	1	1	0	0	1	0	0	1	0	1	0	0	150
1	2	1	0	1	0	1	0	0	1	0	0	0	0
1	3	0	0	0	0	0	0	0	0	0	0	0	0
2	1	1	0	0	1	1	0	0	0	1	0	0	0
2	2	1	1	0	0	0	1	0	0	0	0	1	450
2	3	0	0	0	0	0	0	0	0	0	0	0	0
3	1	1	1	0	0	0	0	0	1	0	1	0	450
3	2	1	0	0	0	1	1	0	0	0	0	1	350
3	3	0	0	0	0	0	0	0	0	0	0	0	0
4	1	1	0	0	1	0	0	0	0	0	0	1	450
4	2	1	1	0	0	1	0	0	1	1	0	0	150
4	3	0	0	0	0	0	0	0	0	0	0	0	0
5	1	1	0	0	0	0	1	0	0	0	0	0	0
5	2	1	0	1	0	1	0	0	0	0	0	1	250
5	3	0	0	0	0	0	0	0	0	0	0	0	0
6	1	1	1	0	0	1	1	0	0	0	1	0	250
6	2	1	0	1	0	0	0	0	1	0	0	1	150
6	3	0	0	0	0	0	0	0	0	0	0	0	0
7	1	1	0	0	1	0	1	0	0	0	0	1	0
7	2	1	0	0	0	1	0	0	0	0	0	0	450
7	3	0	0	0	0	0	0	0	0	0	0	0	0
8	1	1	1	0	0	0	0	0	0	0	1	0	150
8	2	1	0	1	0	1	0	1	0	1	0	0	450
8	3	0	0	0	0	0	0	0	0	0	0	0	0
9	1	1	0	0	0	1	1	0	0	0	1	0	450

Choice task	Alternative	Constant	Facility levels			Facility ownership	Travel mode			Travel time			Out-of-pocket cost
		A	B1	B2	B3	C1	D1	D2	D3	E1	E2	E3	F
9	2	1	1	0	0	0	0	1	0	0	0	0	350
9	3	0	0	0	0	0	0	0	0	0	0	0	0
10	1	1	0	0	1	1	1	0	0	0	0	0	150
10	2	1	0	0	0	0	0	0	1	1	0	0	350
10	3	0	0	0	0	0	0	0	0	0	0	0	0
11	1	1	0	1	0	0	0	0	0	1	0	0	250
11	2	1	0	0	1	1	0	0	1	0	1	0	350
11	3	0	0	0	0	0	0	0	0	0	0	0	0
12	1	1	1	0	0	1	0	0	0	1	0	0	450
12	2	1	0	0	0	0	0	1	0	0	1	0	250
12	3	0	0	0	0	0	0	0	0	0	0	0	0
13	1	1	0	1	0	0	1	0	0	0	1	0	350
13	2	1	0	0	0	1	0	1	0	0	0	1	0
13	3	0	0	0	0	0	0	0	0	0	0	0	0
14	1	1	0	0	1	0	0	0	1	0	0	0	250
14	2	1	0	1	0	1	0	1	0	0	1	0	0
14	3	0	0	0	0	0	0	0	0	0	0	0	0

Note. refer to Esther W. de Bekker-Grob's design. (de Bekker-Grob EW, Donkers B, Jonker MF, Stolk EA. Sample Size Requirements for Discrete-Choice Experiments in Healthcare: a Practical Guide. Patient. 2015 Oct;8(5):373-84.)

Table S3. Selection criteria of latent classes

Classes	LLF	Nparam	CAIC	BIC
2	-25337.72	23	50878.66	50855.66
3	-23422.89	35	47155.03	47120.03
4	-22996.01	47	46407.30	46360.30
5	-21978.28	59	44477.87	44418.87
6	-21587.67	71	43802.67	43731.67
7	-21443.06	83	43619.48	43536.48

Note. LLF, likelihood function; CAIC, Consistent Akaike Information Criterion; BIC, Bayesian Information Criterion.

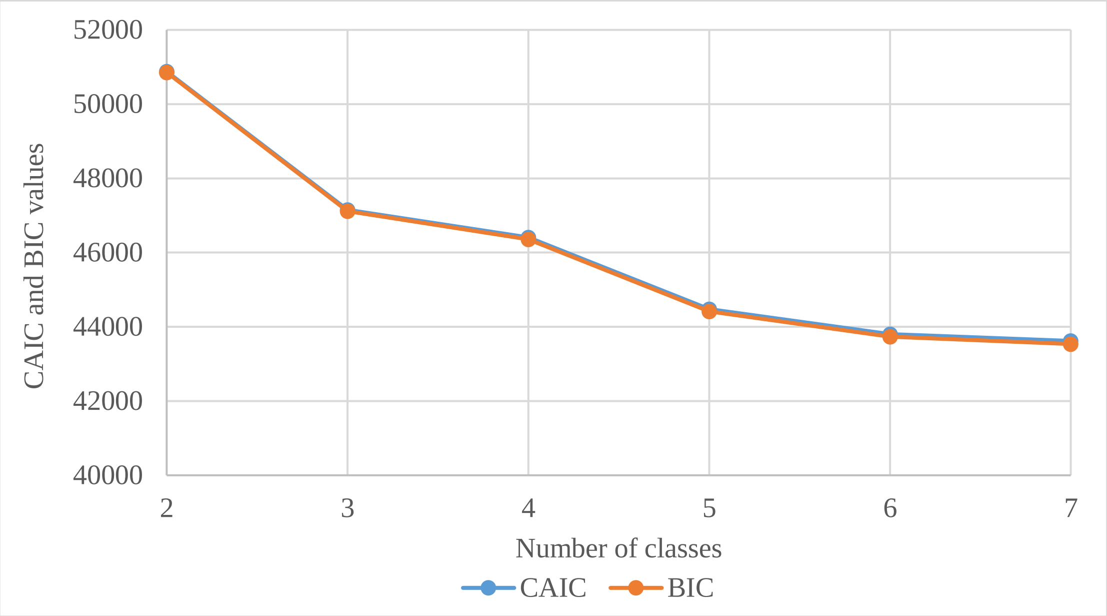

Figure S1. The information criteria (CAIC and BIC) used in determining number of the classes in the latent class logit model

Note. CAIC, Consistent Akaike Information Criterion; BIC, Bayesian Information Criterion.

The CAIC and BIC values of the fifth class are at the inflection point from a sharp decline to a flattening, so the suggested optional number of groups in the latent classes is five.
